# Using Footpad Sculpturing to Enhance the Maneuverability and Speed of a Robotic Marangoni Surfer

**DOI:** 10.3390/biomimetics8050440

**Published:** 2023-09-20

**Authors:** Samuel Bechard, Mitchel L. Timm, Hassan Masoud, Jonathan P. Rothstein

**Affiliations:** 1 Department of Mechanical and Industrial Engineering, University of Massachusetts, Amherst, MA 01003, USA; samuelbechar@umass.edu; 2 Department of Mechanical Engineering-Engineering Mechanics, Michigan Technological University, Houghton, MI 49931, USA; mltimm@mtu.edu (M.L.T.); hmasoud@mtu.edu (H.M.)

**Keywords:** Marangoni effect, surfing robot, remotely controlled, self-powered

## Abstract

From insects to arachnids to bacteria, the surfaces of lakes and ponds are teaming with life. Many modes of locomotion are employed by these organisms to navigate along the air–water interface, including the use of lipid-laden excretions that can locally change the surface tension of the water and induce a Marangoni flow. In this paper, we improved the speed and maneuverability of a miniature remote-controlled robot that mimics insect locomotion using an onboard tank of isopropyl alcohol and a series of servomotors to control both the rate and location of alcohol release to both propel and steer the robot across the water. Here, we studied the effect of a series of design changes to the foam rubber footpads, which float the robot and are integral in efficiently converting the alcohol-induced surface tension gradients into propulsive forces and effective maneuvering. Two designs were studied: a two-footpad design and a single-footpad design. In the case of two footpads, the gap between the two footpads was varied to investigate its impact on straight-line speed, propulsion efficiency, and maneuverability. An optimal design was found with a small but finite gap between the two pads of 7.5 mm. In the second design, a single footpad without a central gap was studied. This footpad had a rectangular cut-out in the rear to capture the alcohol. Footpads with wider and shallower cut-outs were found to optimize efficiency. This observation was reinforced by the predictions of a simple theoretical mechanical model. Overall, the optimized single-footpad robot outperformed the two-footpad robot, producing a 30% improvement in speed and a 400% improvement in maneuverability.

## 1. Introduction

The surfaces of lakes and ponds are teaming with life. Organisms from bacteria to insects to arachnids live on top of the water using surface tension to support their weight and the forces needed for locomotion [1,2,3,4,5,6,7,8,9,10,11]. In most cases, walking, skating, and even jumping are achieved through either a slow, coordinated rowing motion or a rapid spring-like motion of hydrophobic or even superhydrophobic legs or appendages [9,10,12,13]. A few organisms have evolved the unique ability to locally reduce the surface tension of the water they live on by releasing lipid-laden excretions from their anus [9,14]. The resulting gradient in interfacial tension (high in front and low in back) produces a force imbalance that is utilized to propel the organism forward across the surface of the water with surprising speed and dexterity [9,14]. Known as Marangoni propulsion, this phenomenon, which generates interfacial thrust through surface tension gradients, was discovered more than a century ago [15] and has been studied quite extensively in the recent literature both in living creatures and for inanimate objects [9,14,16,17,18,19,20].

These unique forms of propulsion utilized by organisms that live at the air–water interface have recently begun to be implemented in a series of biomimetic water-walking robot designs [13,21]. Most of these robot designs are mechanically driven and use oar-like legs to row across the surface of the water [22,23,24,25]. However, a number of designs have used Marangoni propulsion through the controlled release of surface tension-altering chemicals like alcohols and surfactants to create soap boats or other designs that more closely mimic water-walking insects or arachnids [15,26,27,28,29,30,31,32,33,34,35,36,37,38].

In this study, we will focus on the development and optimization of a remote-controlled Marangoni surfer robot that stands on the surface of the water and uses the controlled release of isopropyl alcohol (IPA) to modify the interfacial tension of water to provide both thrust and steering. We initially introduced this design in Timm et al. [39]. In the current study, we have sculpted and modified the footpads used by the Marangoni robot for floatation to improve the robot’s efficiency, speed, and maneuverability. The design of this robot has an advantage over most other Marangoni surfer robots in the literature, as the vast majority of those robots are self-powered but move in an uncontrolled fashion. Take, for example, the common soap or camphor boats often used as a demonstration of surface tension in high school physics classes [33] or other similar boat-like designs [15,26,27,29,36]. In these examples, the boat is completely uncontrolled as the release rate of the surface tension-altering chemical is not controlled, nor is the boat actively steered. A number of groups have used external sources to affect both release rate and direction of motion. In one example, lasers are used to induce thermocapillary effects that locally change the surface tension and induce motion through Marangoni propulsion [34,40]. These lasers can be moved along the surface of the water to drive motion and steer the Marangoni surfers. More recently, the group of Kwak et al. [30,31,32,41] has developed a series of Marangoni surfer robots that are self-powered and have remote steering capabilities but unlike our design discussed here, they lack active directional and/or speed control.

**Figure 1:**
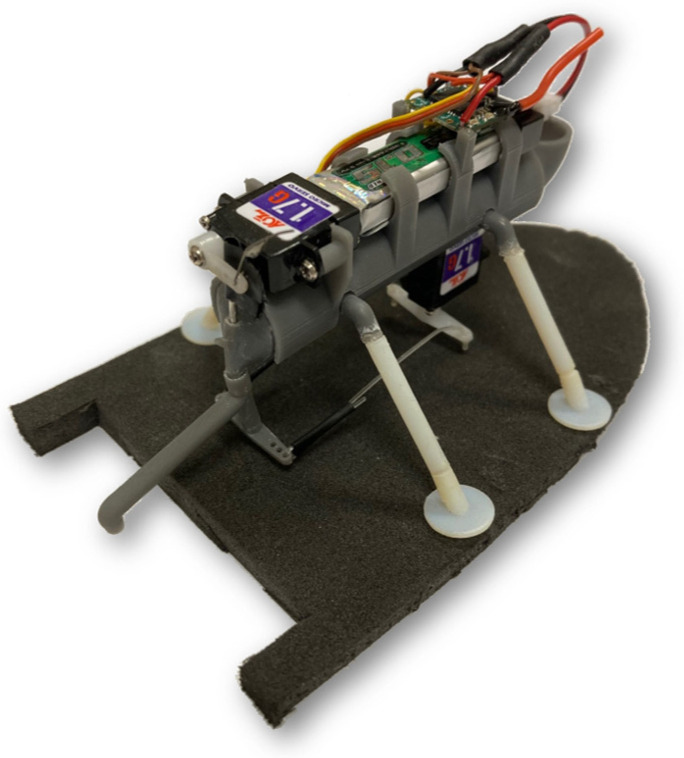
Single footpad design of the remote-controlled Marangoni surfer robot.

The initial design of our remote-controlled Marangoni surfer robot floated on two foam pads and was self-powered using onboard batteries that power two remote-controlled servo motors. A single footpad version of the robot developed here is presented in Figure 1. The first servo motor controlled the location of a needle valve to dictate the release rate of IPA from an onboard IPA reservoir tank. The release rate of IPA was shown to effectively control the swimming speed of the Marangoni surfer robot between 20 mm/s and 100 mm/s. The second servomotor controlled the location of a swivel arm in order to change the side-to-side location where the IPA is released. When released directly behind the robot, a straight-ahead translational motion was produced. When the IPA was released off of the centerline, the resulting interfacial tension gradient was shown to induce a torque on the robot that was capable of efficiently steering the robot around obstacles roughly the size of the surfer with a maximum centripetal acceleration of nearly 20 mm/s^2^. One major benefit of Timm et al.’s design [39] was, unlike other designs that steer through drag-based means like oars or rudders, using Marangoni propulsion to both propel and steer, which significantly increased the efficiency of motion and reduced the cost of transport (CoT) [13,42].

Marangoni surfer robots like these can be used in a host of different applications. With the addition of a camera, these robots can photograph wildlife and study their behavior and habits, monitor bodies of water for invasive species, or even be used for surveillance. All these applications require a long lifetime, easy maneuverability, and efficient propulsion. To address these design criteria, in this study, we investigate several modifications to the design of the foam footpads with the goal of decreasing the CoT while maintaining or improving the maneuverability of our Marangoni surfer robot. These changes included a variation in the gap between the original two individual footpads and replacing the two footpads with a single footpad of the same surface area with a sculptured backside to capture and efficiently direct the surfactant-induced Marangoni forces.

## 2. Robot Design

**Figure 2:**
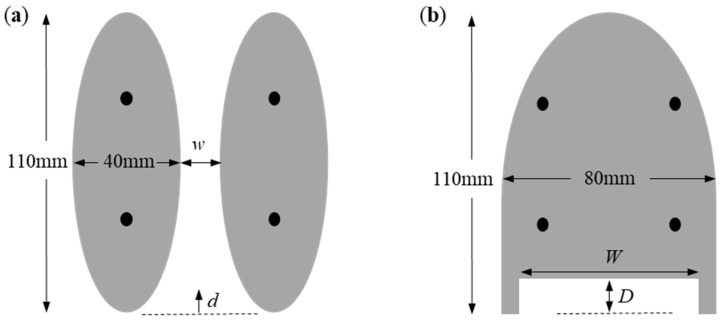
Modified designs of the floatation footpads of the remote-controlled Marangoni surfer robot. In (**a**), two elliptical footpads separated by a variable distance *w* were studied with an IPA released at location *d* = 0 mm. In (**b**), a single footpad with an elliptical leading edge and a rectangular cut-out having variable width, *W*, and depth, *D*, was studied. The locations of the robot’s legs are shown as black circles.

A complete description of the robot surfer design can be found in Timm et al. [39]. For completeness, we will provide an overview of the robot design here. As seen in Figure 1, the body of the surfer, which weighs roughly 18 g, doubles as both the storage vessel for the IPA propellant and the mounting point for the servos, batteries, control mechanism, and radio receiver for the remote control. The 3D printed tank has a capacity of 4 mL of IPA that was shown to supply between 30 s and 5 min of continuous propulsion depending on the commanded position of the needle valve. The IPA was released via a gravity feed through an extension tube shown in Figure 1 that, when aligned along the centerline of the surfer body, released IPA in line with the rear edge of the floating footpads (*d* = 0 mm) as shown in Figure 2a. Steering was achieved using a servo motor to swivel the extension tube left or right during the release of IPA. The maximum rotation was 10 mm off the centerline. The remote control for both steering and speed was implemented using a micro-receiver (OrangeRx R614XN) and a remote transmitter (Spektrum DX7s) connected to two micro-servo motors (HobbyKing HK-5320s). Both the motors and the controller were powered by a miniature onboard battery (Turnigy Nano-Tech 150 mAh). All structural elements were designed with sufficient structural rigidity such that no deformation or deflection of components was observed during operation.

The footpads were fabricated from a rigid polyurethane foam (McMaster–Carr) with a density of 32 kg/m^3^ and a modulus of roughly 50 MPa. The footpads were not observed to deform or deflect under the weight of the robot or the forces experienced during surfing. The footpads were designed to provide the necessary buoyancy to safely float the 18 g robotic surfer with a 5× factor of safety while also producing low hydrodynamic drag and maintaining a design/shape that mimicked a water-walking insect. As a design constraint for this study, the overall area of the footpads and, therefore, the buoyancy force were held roughly constant so that comparisons between different footpad designs could be more easily made. The original footpads were elongated ellipses with a major axis of 110 mm and a minor axis of 40 mm and were 10 mm thick. When mounted on the robot’s legs, the two pads produced a catamaran design with a gap of 5 mm between the two footpads. Although the design of the footpads was initially chosen to provide a reduced hydrodynamic drag, the gap between the two footpads had a negative effect on propulsion. The gap allowed the IPA that was released at the rear of the robot to be drawn forward between the two footpads by the higher interfacial tension in the clean water ahead of the robot surfer. The result was a loss of potential thrust and a reduced overall propulsion efficiency. In this study, we investigated the influence of the footpad separation on robot speed and maneuverability through a series of experiments performed with footpads mounted with an increasing separation distance that varied between 0 mm < *w* < 30 mm. In addition to the two-footpad catamaran design shown in Figure 2a, a single-footpad design was also tested with no separation between pads. In this design, shown in Figure 2b, the leading edge of the footpad was derived from an ellipse with a major axis of 110 mm and a minor axis of 70 mm. From the midpoint of the ellipse, the rear of the footpad was then extended straight back to produce a footpad with a final length of 110 mm. At the rear of the footpad, a rectangular cut-out was introduced to efficiently capture the IPA and maximize the duration that the interfacial tension is reduced in the wake by a single drop of IPA. Both the depth and width of the cut-out were varied between 5 mm < *D* < 15 mm and 36 mm < *W* < 56 mm, respectively, with the goal of increasing the efficiency of propulsion and the maneuverability of the surfer.

## 3. Experiments

**Figure 3:**
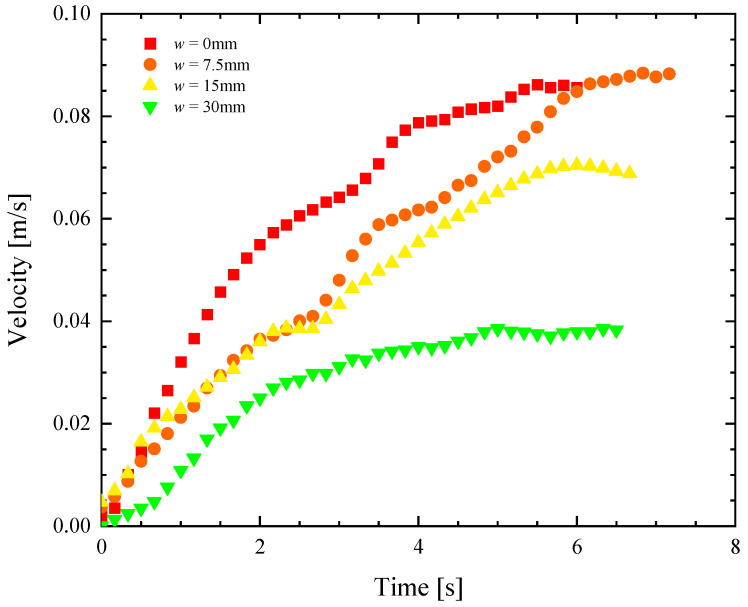
Velocity as a function of time for a remote-controlled Marangoni surfer robot with two elliptical footpads. The data include footpads with separation of (■) *w* = 0 mm, (●) *w* = 7.5 mm, (▲) *w* = 15 mm and (▼) *w* = 30 mm.

**Figure 4:**
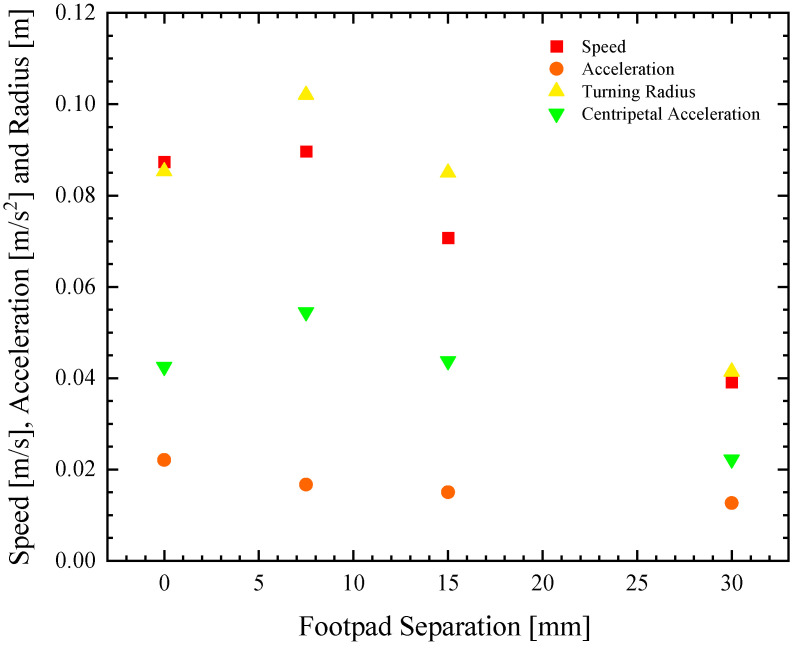
Speed and maneuverability data as a function of footpad separation for a remote-controlled Marangoni surfer robot with two elliptical footpads. The data include (■) maximum linear speed (●) initial acceleration, (▲) turning radius, and (▼) centripetal acceleration.

**Figure 5:**
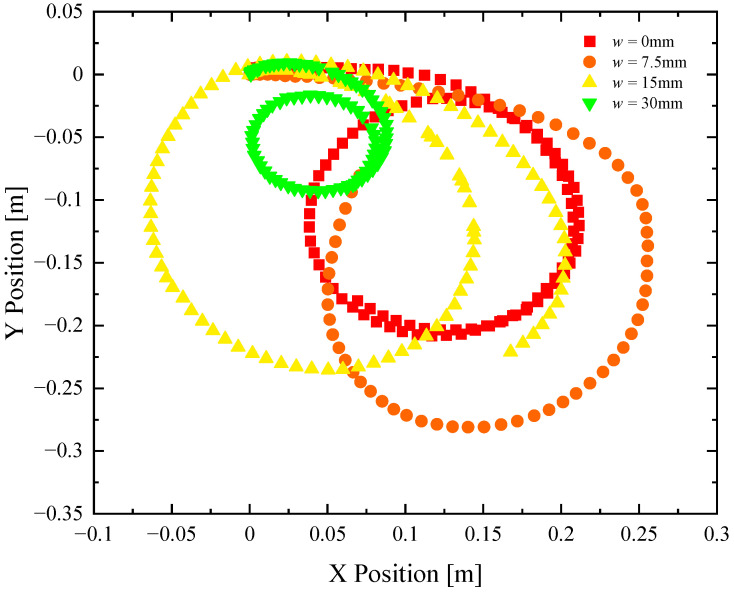
Trajectories of a remote-controlled Marangoni surfer robot with two elliptical footpads executing a turn at maximum throttle. The X- and Y-position data include footpads with separation of (■) *w* = 0 mm, (●) *w* = 7.5 mm, (▲) *w* = 15 mm, and (▼) *w* = 30 mm.

**Figure 6:**
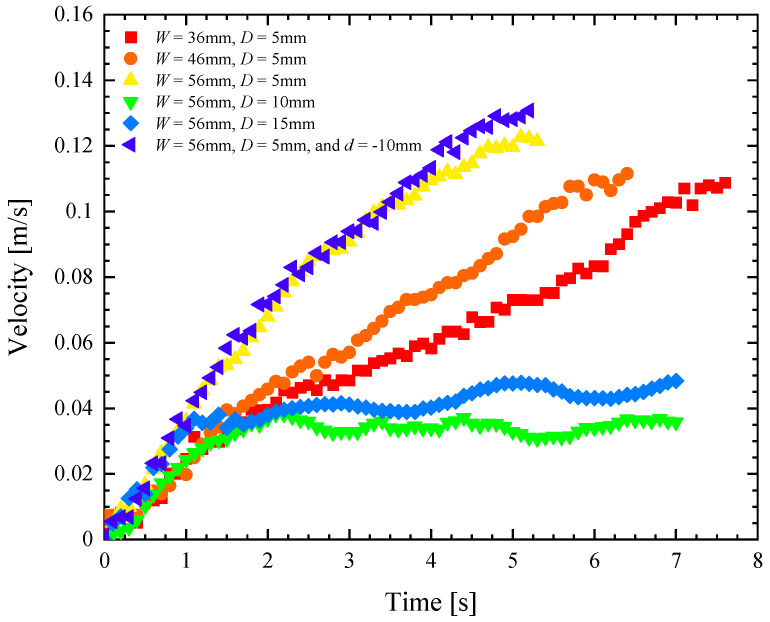
Velocity as a function of time for a remote-controlled Marangoni surfer robot with a single footpad with an elliptical leading edge and a trailing edge with rectangular cut-out having variable width, *W*, and depth, *D*. The data include cut-outs with (■) *W* = 36 mm and *D* = 5 mm, (●) *W* = 46 mm and *D* = 5 mm, (▲) *W* = 56 mm and *D* = 5 mm, (▼) *W* = 56 mm and *D* = 10 mm, (♦) *W* = 56 mm and *D* = 15 mm, and (◄) *W* = 56 mm, *D* = 5 mm, and *d* = −10 mm.

**Figure 7:**
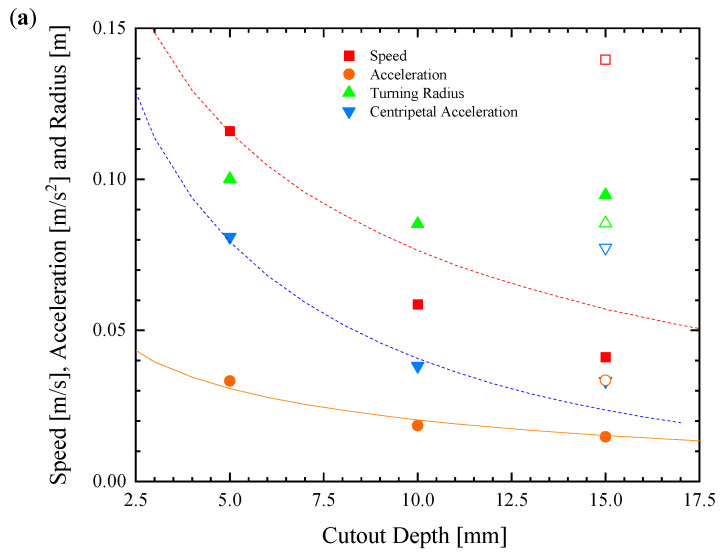

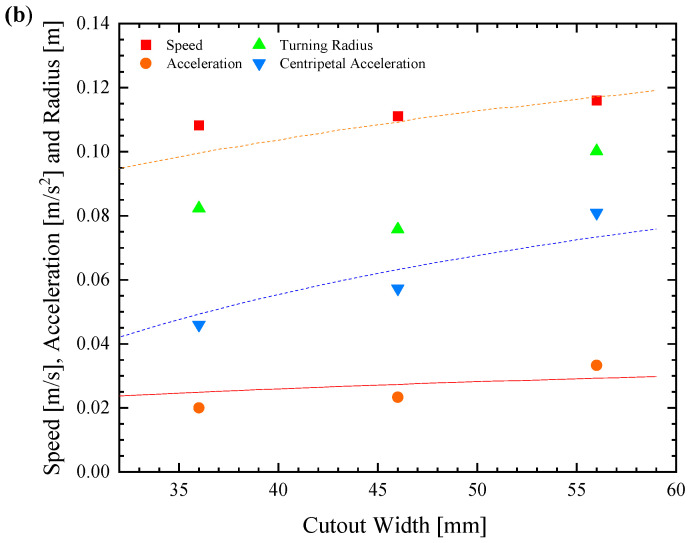
Speed and maneuverability data for the remote-controlled Marangoni surfer with a single footpad with varying cut-out depth at a constant width of *W* = 56 mm (**a**) and cut-out width at a constant depth of *D* = 5 mm (**b**). The data include (■) speed, (●) linear acceleration, (▲) turning radius, and (▼) centripetal acceleration. Filled symbols in (**a**) correspond to *d* = 0 mm, while hollow symbols are for *d* = −10 mm. Lines from theoretical predictions are superimposed over the data.

To assess the performance of our remote-controlled Marangoni surfer robot, we performed two tests. The first experiment tested the acceleration and top straight-line speed of the robot. The Marangoni robot was placed on the surface of water 0.1 m from the edge of a rectangular acrylic tank measuring 1.0 m by 0.5 m. The tank was filled to a depth of 0.25 m with clean water for each experiment. The throttle was set wide open, producing a volume flow rate of IPA of *q* = 0.15 mL/s. A USB camera was used to capture the motion of the Marangoni robot at 30 fps as it accelerated from rest and reached a steady state velocity. The video was digitized using the free software Tracker, and the velocity and linear acceleration were numerically calculated from the position versus time data. The experiment was stopped either when the robot ran out of fuel or made contact with the far wall of the tank. A minimum of three independent experiments were performed for each configuration to ensure repeatability. When plotting robot trajectories like in Figure 3, Figure 5 and Figure 6, representational data sets are used. When plotting data like maximum velocity, acceleration, and turning radius, like in Figure 4 and Figure 7, average values over all the data sets are presented. The second experiment tested the maneuverability of the Marangoni robot. In this test, the robot was placed on the surface of the water 0.5 m from the back wall and 0.1 m from the left wall of the tank. The throttle was set to maximum, and the extension tube was swiveled all the way to the left (10 mm displacement) in order to execute a hard right turn at high speed. The motion of the Marangoni robot was again captured and digitized to determine the velocity, turning radius, and centripetal acceleration of the robots with different footpad designs. The turning radius was determined by fitting a circle to the Marangoni robot’s trajectory.

## 4. Results and Discussion

### 4.1. Two-Footpad Design

The effect of separation between the two footpads shown in Figure 2a was investigated for Marangoni surfer robots with footpad separations of *w* = 0, 6, 12, and 30 mm. The effect of release location, *d*, was studied by Timm et al. [39]. They showed that the velocity increased as the IPA was released closer to the footpads (*d* became less positive), but they also showed that the in-line motion of the Marangoni robot became unstable if the IPA was released between the two pads, *d* < 0. For that reason, we chose to release the IPA in line with the trailing edge of the footpads at *d* = 0 mm.

In Figure 3, representational velocity profiles as a function of time are presented for the four different pad separations as they accelerate from rest. The data, down-sampled here from 30 fps to 5 Hz for clarity, show a clear difference in the response as the pad separation is increased. Increasing the footpad separation not only reduces the maximum velocity attained by the robot surfer but also reduces the initial acceleration. The average values for the speed and the acceleration over all the experiments at each test condition are also plotted in Figure 4 for an easier comparison of the trends. As seen in Figure 4, the speed and the initial acceleration of the robot decreased by more than 50% as the footpad separation was increased from *w* = 0 to 30 mm. Interestingly, even though the initial acceleration decreased by 25% when the separation was increased from 0 to 7.5 mm, the maximum velocity actually increased slightly. The initial acceleration from rest can be easily converted to a thrust force, $F_{T}=ma$, as long as one knows the mass of the Marangoni robot, *m* = 18 g. This is because, during the early stages of acceleration from rest, the forces associated with the acceleration of the robot are significantly larger than hydrodynamic drag forces. It is at larger speeds where the Marangoni robot reaches a steady state velocity, where the hydrodynamic drag dominates. Thus, the thrust force can be calculated from early time data, and the drag coefficient of the Marangoni surfer can be calculated at later times because Fdrag=12cdρU2Af. Here, *ρ* is the fluid density, *c_d_* is the drag coefficient, *U* is the robot velocity, and *A_f_* is the frontal area of the robot. A closed gap of *w* = 0 mm produces roughly 25% more thrust force on the Marangoni robot by limiting the flow of fluid upstream through the gap between the two footpads induced by the gradient in surface tension. However, the data also show that the two-footpad design has a 25% smaller drag coefficient when the gap between the pads is increased to *w* = 7.5 mm. Thus, the Marangoni robot’s straight-line performance is a non-monotonic function of the footpad spacing with optimal performance when the footpads have a small, non-zero spacing of *w* = 7.5 mm. As we will see, similar observations were made for maneuverability, although the conclusions are perhaps a little more intuitive as spreading the footpads apart provides larger moment arms for the production of torque.

In Figure 5, a series of trajectories are plotted for the Marangoni swimmer turning at full throttle, and the extension tube swiveled all the way left (10 mm off centerline) to produce the tightest turn possible. The trajectories show an initial transient response where the Marangoni robot is accelerating into the turn from rest, followed by a longer time response where the Marangoni robot follows a circular path along the surface of the water. The data points are at constant time intervals, so the velocity of the Marangoni robot can be inferred from the separation between data points. The experiments were stopped after the completion of a single circular path to ensure that the surfer was moving through a clean, IPA-free interface throughout the course of the experiment. The turning radius, *R*, was measured for each experiment and footpad spacing. The value of the turning radius was quantified and plotted in Figure 4. The turning radius was found to be a non-monotonic function of footpad spacing, with a very small turning radius at *w* = 30 mm and the largest turning radius at *w* = 7.5 mm. These results would suggest that the Marangoni robot with the largest footpad separation was the most maneuverable. However, turning radius is, in fact, not the best way to understand the maneuverability of the Marangoni surfer. This is because the Marangoni robots were not driven with a constant velocity but at a constant IPA release rate. As a result, each experiment achieved a different velocity, and turning is more difficult at larger speeds. A better way to interpret the maneuverability data is to look at the centripetal acceleration, $a_{c}=U^{2}/R$, which takes into account the instantaneous velocity of the Marangoni robot during the trajectories shown in Figure 5, *U*. Note that this velocity is not the same as the straight-line velocity measured previously in Figure 3 and tabulated in Figure 4. A very different trend can be observed when examining the centripetal acceleration data in Figure 4. Here, we see that the largest centripetal acceleration is achieved with a footpad spacing of *w* = 7.5 mm, the same spacing that showed the largest turning radius. The induced centripetal acceleration for *w* = 7.5 mm is 30% larger than *w* = 0 mm and nearly 150% larger than for *w* = 30 mm. As seen in the linear straight-line motion experiments, increasing the footpad separation to *w* = 7.5 mm slightly improved the straight-line speed of the Marangoni robot. When the large propulsive force is combined with the increased centripetal acceleration, the result is a more maneuverable robot and optimized two-footpad design.

### 4.2. Single Footpad Design

In order to eliminate the loss of IPA through the gap between the two footpads of the original design, a series of single footpad designs were conceived as described in Section 2 and tested both for speed and maneuverability. For the single footpad design, the effect of the width and depth of the rear cut-out seen in Figure 2b were studied while holding everything else constant. Three different depths of *D* = 5, 10, and 15 mm were studied at a constant width of *W* = 56 mm. Additionally, at a depth of *D* = 5 mm, three different widths of *W* = 36, 46, and 56 mm were studied. In all cases but one, the IPA was released at a position of *d* = 0 mm. For the *D* = 15 mm and *W* = 56 mm Marangoni robot, one additional experiment was performed, where the IPA was released well within the cut-out at *d* = −10 mm to determine if performance could be further improved by releasing the IPA closer to the rear edge of the robot and the base of the cut-out.

In Figure 6, a representative data set of straight-line velocity versus time is presented for each of the six different single footpad designs. From these data, several clear trends emerge. First, increasing the depth of the rectangular cut-out was found to adversely affect the speed and initial acceleration of the Marangoni surfer. Perhaps this can be seen better in Figure 7a, where speed, acceleration, turning radius, and centripetal acceleration are plotted as a function of the cut-out depth, *D*. Increasing the depth from *D* = 5 mm to 15 mm decreased the maximum speed of the Marangoni surfer by a factor of nearly 3× while simultaneously decreasing the initial acceleration by more than 50%. This observation is likely due to the fact that as the IPA spreads along the surface of the water, its concentration decays. Numerical simulations of IPA release behind a sphere with a constant mass flux suggest a polynomial decay of the IPA concentration, *c*, with a slope of $c\propto 1/r^{1.2}$, where *r* is the distance from the release point of the IPA [17]. Because the change in surface tension, σ, from pure water is linearly proportional to the IPA concentration, Δσ∝c, the reduction in surface tension, which is responsible for the Marangoni forces that propel the surfer, decays quickly, $\Delta \sigma \propto 1/r^{1.2}$, with increasing distance from the dripping location. This hypothesis was tested by changing the location of IPA release from *d* = 0 mm to *d* = −10 mm for the *W* = 56 mm, *D* = 15 mm case. This setup produces an IPA release that is 5 mm from the trailing edge of the Marangoni robot. If distance and decay in surface tension reduction is the principal variable controlling the motion of the Marangoni robot, it is expected that this case should be essentially equivalent to the *W* = 56 mm, *D* = 5 mm case. In Figure 6 and Figure 7a, it is apparent that these two data sets are essentially superimposed with the *W* = 56 mm, *D* = 5 mm, and *d* = −10 mm case, showing just a slight increase in velocity that is likely due to some additional trapping of IPA in the wake of the Marangoni robot by the side walls of the rectangular cut-out. Further increases in velocity and acceleration are likely possible with an even closer IPA release. A simple theory will be presented in Section 4.3 to capture the scaling of the speed and acceleration of the Marangoni robot with the width and depth of the cut-out and the release location of the IPA.

The second clear trend is that the width of the rectangular cut-out was found to increase both the initial acceleration of the Marangoni surfer as well as the maximum velocity. As seen in Figure 6 and Figure 7b, increasing the width from 36 mm to 56 mm, a change of more than 50%, resulted only in a roughly 10% increase in the maximum speed of the Marangoni surfer. This is also the result of the radial decay in the surface tension reduction. As a result, the change in surface tension far from the release point is small compared to the change close to the centerline where the distance, $r=\sqrt{{\left(D-d\right)}^{2}+y^{2}}$, is dominated by the depth and distance from the centerline, *y*. Thus, once the width of the cut-out becomes large compared to the depth, further increasing the width of the cut-out will not increase the surfer’s speed much further.

**Figure 8:**
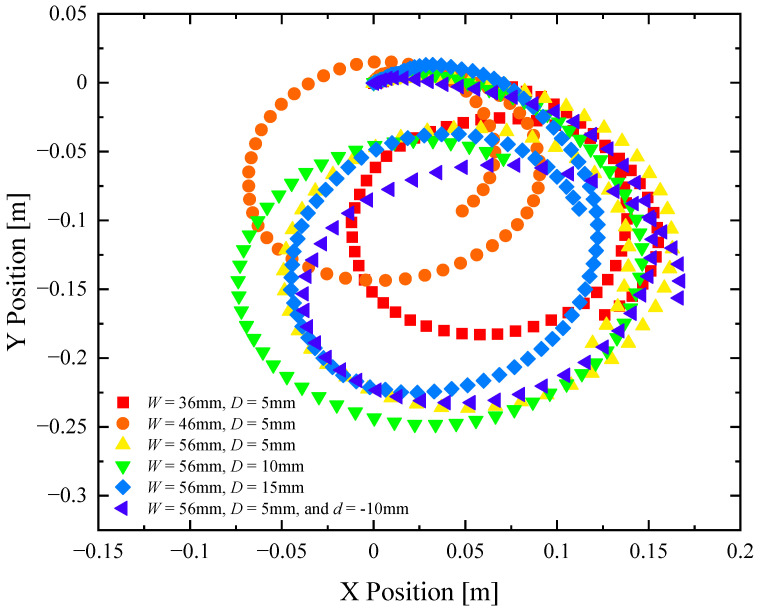
Trajectories from maneuverability experiments of a remote-controlled Marangoni surfer robot with a single footpad. The data include X- and Y-position data for designs having cut-outs with (■) *W* = 36 mm and *D* = 5 mm, (●) *W* = 46 mm and *D* = 5 mm, (▲) *W* = 56 mm and *D* = 5 mm, (▼) *W* = 56 mm and *D* = 10 mm, (♦) *W* = 56 mm and *D* = 15 mm, and (◄) *W* = 56 mm, *D* = 5 mm, and *d* = 10 mm.

The same is not true when it comes to maneuverability. In Figure 8, a series of trajectories are plotted for the robot turning at the maximum throttle and swivel of the extension tube in order to execute a high-speed turn. From Figure 7b, no clear trend in the turning radius with cut-out width was found. Again, this is because the Marangoni robots were not driven with a constant velocity but at a constant IPA release rate. The centripetal acceleration, again, is a better measure of the Marangoni robot’s maneuverability. Here, a clear trend can be observed in Figure 7. Increasing the depth of the cut-out from 5 mm to 15 mm resulted in more than a 50% reduction in centripetal acceleration while increasing the width of the cut-out from 36 mm to 56 mm, a 75% increase in the centripetal acceleration of the Marangoni surfer was observed. The latter observation is because even though the concentration of IPA decays with distance from the deposition location, the moment arm of the applied torque increases as one moves off of the centerline of the Marangoni surfer. Moreover, unlike the case of two footpads, thrust is not lost with increasing cut-out width as it was with increasing footpad spacing. Thus, even small forces applied far from the centerline can impose a large torque, producing the large centripetal acceleration seen here. For comparison, the single footpad design with a 56 mm wide and 5 mm deep cut-out produced a nearly 400% improvement in centripetal acceleration compared to the initial design of Timm et al. [39] and a 150% improvement over the best two-footpad design presented here. When coupled with the 30% increase in maximum velocity, it is clear that changing from a two-pad to a single-pad design had a significant impact on both the speed and maneuverability of the Marangoni robot.

### 4.3. Theory

**Figure 9:**
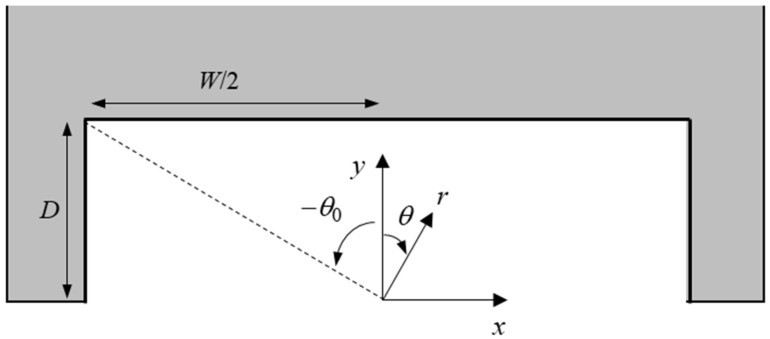
Schematic diagram showing the geometry of the rear of the single footpad robot and the location of the cut-out.

To better understand the trends in the results, a simple theory was proposed. In Figure 9, a schematic diagram of the cut-out in the rear of the single footpad Marangoni robot is shown with the appropriate geometry labeled. Unfortunately, modeling the two-footpad Marangoni robot was not possible with this simplified approach and will require a full computation fluid dynamics treatment in the future. In order to determine the variation in the Marangoni force and imposed torque with variations in depth and width, the force across the rear and side walls of the cut-out was calculated after making a few simple approximations. First, it was assumed that the surface tension decayed with one over the radius to the 1.2 power such that, $\gamma =\gamma_{w}-c_{1}/r^{1.2}$. Here, $\gamma_{w}$ is the surface tension of clean water, and *c*_1_ is a constant that will be determined by fitting the results in Figure 7. This assumes that the IPA spreads radially outward from the drop location on the surface of the water without significant diffusion into the bulk, as predicted by Kang et al. [17]. In order to calculate the variation in the propulsion force as a function of time, the interfacial tension was numerically integrated along the back surface of the cut-out according to

(1) $$F_{y}=\int_{-W/2}^{W/2}\left(\gamma_{w}-\gamma \right)dx=\int_{-W/2}^{W/2}\frac{c_{1}}{{\left(D^{2}+x^{2}\right)}^{0.6}}dx.$$

Unfortunately, the scaling with *r* did not lead to a nice closed-form solution. The results of numerically integrating Equation (1) are superimposed over the data in Figure 7. The value of *c*_1_ was chosen to obtain the best fit to the linear acceleration data set using a surfer mass of 18 g.

The force data can also be used to predict the maximum surfer speed. Here, it was assumed that the majority of the drag force came from the laminar boundary layer on the flat bottom of the footpad. The Reynolds number was calculated to be quite large, Re=ρUL/μ≈103−104, but not large enough to cause the boundary layer to become turbulent. Here, *ρ* is the water density, *U* is the robot velocity, *L* is the length of the footpad, and *μ* is the viscosity of water. As a result, from Blasius boundary layer theory, the drag force scales as $F_{drag}=c_{2}U^{3/2}$, [43] where *c*_2_ is a constant that includes information about the surface area, the viscosity, and the density of the water, all of which are constant for the Marangoni robot so they can be lumped into a single constant and fit to the data in Figure 7. With the constant fit, the maximum robot scales with the square root of the propulsive force, $U_{\max}={\left(F_{y}/c_{2}\right)}^{2/3}$. The results of theoretical predictions are superimposed over the data. The theory does a good job of qualitatively predicting the trends in maximum velocity data.

In order to better understand the effect of the cut-out width and depth on the Marangoni robot’s maneuverability, the toque applied to the surfer by the off-center release of the IPA was calculated according to

(2) $$T=\int_{-W/2}^{W/2}F_{y}\;x\;dx+\int_{0}^{D}F_{x,L}\;y\;dx+\int_{0}^{D}F_{x,R}\;y\;dx.$$

Here, we assume that the IPA is released a distance, *L_x_*, off the centerline and the torque is produced on three surfaces. The two side walls provide torques in opposite directions, and the back wall provides a torque due to the asymmetric IPA distribution and surface tension along the back wall. The resulting equation was again solved numerically. A number of interesting observations can be made. Increasing the distance from the centerline that the IPA was released resulted in a roughly linear increase in the torque. Additionally, the majority of the torque was produced not by the side walls of the cut-out, but by the surface tension variation along the back surface of the cut-out. This was unexpected given the large moment arm of the sidewall, but it was found that the fast decay of the IPA concentration resulted in much smaller Marangoni forces along the sidewalls. The results of the theory are superimposed over the centripetal acceleration in Figure 7 and are found to predict the scaling of the data with depth and width quite well.

## 5. Conclusions

In this paper, we drew motivation from insects and arachnids that live on the air–water interface to design, develop, and optimize a remote-controlled robotic platform that uses surface tension variations to both efficiently propel and steer itself along the surface of the water. This biomimetic robot used an onboard tank of IPA and a series of servo motors powered by onboard batteries and controlled by a remote transmitter and receiver to both meter out the propellant and steer the robot. Steering was achieved by releasing the IPA off the centerline axis to introduce an asymmetry in the surface tension to produce torque on the robot. The original design of this Marangoni robot was presented by Timm et al. [39]. Here, we studied the effect of a series of modifications to both single and double footpad designs.

These footpads, which are necessary to float the robot, are also integral to efficiently converting the surface tension gradients produced by the addition of IPA in the wake of the robot into propulsive force and steering torques. For the two-footpad design, the effect of changing the separation between the two footpads was investigated. It was shown that a small but non-zero gap of *w* = 7.5 mm had the best combination of performance characteristics with the largest maximum velocity, strong linear acceleration, and the best centripetal acceleration during turn tests of all the designs studied. Increasing the separation past *w* > 7.5 mm was found to have a negative impact on both the robot’s efficiency and maneuverability due to the loss of IPA pulled upstream through the gap between the two footpads. The single footpad design eliminated this problem. It was shown that single footpads with wider and shallower cut-outs helped contain the released IPA and increase the efficiency of propulsion and maneuverability. Overall, the single-footpad design was found to be superior to the two-footpad design, with a nearly 400% improvement in centripetal acceleration and a 30% increase in maximum speed compared to the initial design of Timm et al. [39]. Our optimized Maragnoni robot design also outperforms the robots of Kwak and Bae [31,32] that have the most closely related functionality and form to ours.

## Data Availability

Data is available by request from authors.
